# Metabolite Profiling of *Alangium salviifolium* Bark Using Advanced LC/MS and GC/Q-TOFTechnology

**DOI:** 10.3390/cells10010001

**Published:** 2020-12-22

**Authors:** Chandranayaka Siddaiah, Anil Kumar BM, Saligrama Adavigowda Deepak, Syed Salman Lateef, Saurabh Nagpal, Kanchugarakoppal S. Rangappa, Chakrabhavi D. Mohan, Shobith Rangappa, Madan Kumar S, Minaxi Sharma, Vijai Kumar Gupta

**Affiliations:** 1 Department of Studies in Biotechnology, University of Mysore, Manasagangotri, Mysore 570006, India; anilkumarbm0908@gmail.com; 2 Agilent Technologies India Pvt. Ltd., Bangalore 560048, India; deepak_sa@agilent.com (S.A.D.); syed.lateef1@wipro.com (S.S.L.); saurabh_nagpal@agilent.com (S.N.); 3 Wipro Ltd., SEZ, Sarjapura, Bangalore 560099, India; 4 Institution of Excellence, Vijnana Bhavan, University of Mysore, Manasagangotri, Mysore 570006, Inida; rangappaks@gmail.com (K.S.R.); madan.mx@gmail.com (M.K.S.); 5 Department of Studies in Molecular Biology, University of Mysore, Manasagangotri, Mysore 570006, India; cd.mohan@yahoo.com; 6 Adichunchanagiri Institute for Molecular Medicine, AIMS Campus, B. G. Nagar, Mandya 571448, India; shobithrangappa@gmail.com; 7 Department of Food Technology, Akal College of Agriculture, Eternal University, Baru Sahib, Himachal Pradesh 173101, India; minaxi86sharma@gmail.com; 8 Center for Safe and Improved Food, Scotland’s Rural College (SRUC), Kings Buildings, West Mains Road, Edinburgh EH9 3JG, UK; 9 Biorefining and Advanced Materials Research Center, Scotland’s Rural College (SRUC), Kings Buildings, West Mains Road, Edinburgh EH9 3JG, UK

**Keywords:** *Alangium salviifolium*, LC/MS, GC/Q-TOF

## Abstract

There is an urge for traditional herbal remedies as an alternative to modern medicine in treating several ailments. *Alangium salviifolium* is one such plant, used traditionally to treat several diseases. In several reports, there are findings related to the use of this plant extract that demonstrate its therapeutic value. However, very few attempts have been made to identify the extensive metabolite composition of this plant. Here, we performed metabolite profiling and identification from the bark of *A. salviifolium* by extracting the sample in organic and aqueous solvents. The organic and aqueous extracts were fraction-collected using the Agilent 1260 Analytical Scale Fraction Collection System. Each of the fractions was analyzed on Liquid Chromatogaphy/Quadrupole Time-of-Flight LC/Q-TOF and Gas Chromatography/Quadrupole Time-of-Flight GC/instruments. The Liquid Chromatography/Mass Spectrometry (LC/MS) analyses were performed using Hydrophilic Ineraction Liquid Chromatography (HILIC), as well as reversed-phase chromatography using three separate, orthogonal reverse phase columns. Samples were analyzed using an Agilent Jet Stream (AJS) source in both positive and negative ionization modes. The compounds found were flavonoids, fatty acids, sugars, and terpenes. Eighty-one secondary metabolites were identified as having therapeutic potential. The data produced was against the METLIN database using accurate mass and/or MS/MS library matching. Compounds from *Alangium* that could not be identified by database or library matching were subsequently searched against the ChemSpider) database of over 30 million structures using MSMS data and Agilent MSC software.In order to identify compounds generated by GC/MS, the data were searched against the AgilentFiehn GCMS Metabolomics Library as well as the Wiley/NIST libraries.

## 1. Introduction

Medicinal plants have the capacity to produce a variety of chemical compounds that are used to perform important biological functions. Majority of health care products available on the market are known to be derived from plants. Recently, the World Health Organization estimated that 80% of the world population relies on herbal medicines for some aspect of their primary health care needs, and, according to them, around 21,000 plant species have the potential to be used medicinally [1].The use of plant-based medications and therapeutics is continuously increasing worldwide; hence, there is high acceptance and demand [2]. *Alangium salviifolium* (L.f) Wang is a medicinal plant reported in Ayurveda and Chinese medicine. This plant is used traditionally to treat several diseases such as cancer, leprosy, diabetes, paralysis, microbial infections, and others. Plant parts such as roots, stems, leaves, flowers, fruits, or the entire plant extract are consumed orally or applied dermally, depending on the type of disease that is treated. Experiments correlating this medicinal plant with specific diseases or activities were reported earlier [3,4,5,6]. This plant extract shows antiepileptic [7], antioxidant/antimicrobial [8], antidiabetic [9], wound healing [10], antiarthritic [11], antibacterial [12], antifertility [13], cardiac [14], anti-inflammatory [15], diuretic [16], and antifungal [17] effects. Comprehensive untargeted metabolomics provides an unbiased analysis of all biochemical intermediates in a sample. This is achieved by using complementary universal analytical techniques such as LC/MS, GC/MS, and NMR. The factors that can affect the evaluation of a metabolome include the method used for sample harvesting/extraction procedures, fractionation, chromatographic separation chemistry, ionization techniques/modes, acquisition parameters, data processing/analysis, and identification [18]. In this study, by using orthogonal LC/MS and GC/MS techniques, we aimed for a comprehensive analysis, including identification of the metabolites present in stem bark for this plant. 

## 2. Materials and Methods

### 2.1. Reagents and Materials

LC/MS grade isopropanol, methanol, and acetonitrile were purchased from Fluka (Germany). Milli Q water (Millipore Elix 10 model, Darmstadt, Germany) was used for mobile phase preparation. The additives, namely ammonium fluoride, acetic acid, ammonium formate, formic acid, and ammonium acetate, were procured from Fluka (Germany).

### 2.2. Workflow

**Figure 1:**
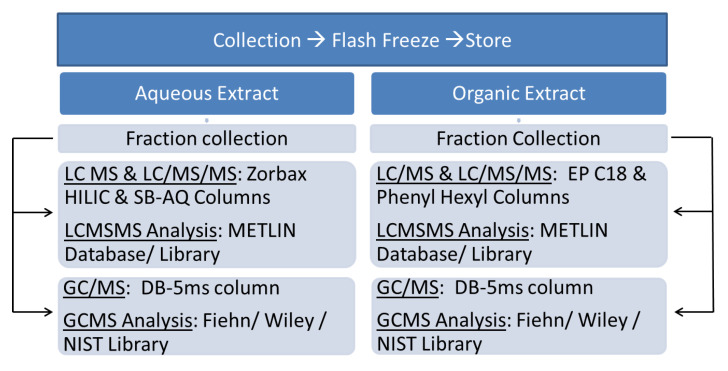
Summary of the workflow for biphasic solvent extraction followed by analysis using LC/MS and GC/MS platforms.

The workflow followed for this study is outlined in Figure 1. 

### 2.3. Collection of Plant Material and Extraction Procedure

Bark of *A. salviifolium* was collected from the plants near Mysore, India, and immediately transferred to liquid nitrogen and stored in −80 °C until further use. Two grams of bark tissue was powdered using a mortar and pestle in the presence of liquid nitrogen. For extraction, 40 mL of degassed solution containing chloroform:methanol:water in the ratio of 1:2.5:1 (*v*/*v*/*v*) was added. The solution was crushed for 5 min, transferred to 1.5 mL eppendorf tubes, and vortexed for 5 min at 4 °C. The tubes were centrifuged at 20,800× *g* for 2 min, and the supernatant was pooled from all the tubes into a glass vial. One milliliter of the supernatant was transferred to the eppendorf tube, and 400 μL of water was added. The tubes were vortexed for 10 s, followed by centrifugation at 20,800× *g* for 2 min. The aqueous (upper) and organic (lower) layers were separated and dried separately in a speed vac (Eppendorf, Hamburg, Germany).

### 2.4. Fraction Collection

To the dried aqueous and organic layers, 200 μL of 50:50 and 30:70 of mobile phase A and B of respective fractionation method (see Table 1) were added. The vials were sonicated to resuspend the compounds. HPLC separation was performed by injecting the resuspended mixtures from 5 vials to an Agilent 1, (Santaclara, CA, USA) 260 Infinity analytical purification system equipped with a 1 mL Manual FL-Injection valve (p/n: 5067-4191).The fractionswerecollected in 45 wells of a 96-well plate and dried in a speed vac. 

### 2.5. Multiple LC/Q-TOF Chromatographic Analysis Conditions

The dried aqueous fractions were resuspended in 250 μL of 50:50 of methanol:water containing 0.2% acetic acid and sonicated for 10 s, whereas the organic fractionsweresuspended in 30:70 of mobile phase A and B (Table 2—organic), followed by centrifugation at 3000 rpm for 10 min. Five microliters of the resuspended fractionswas injected into an Agilent 1260 Infinity LC System, interfaced to an Agilent 6540 accurate mass Q-TOF LC/MS system (Santa clara, CA, USA). The reference solution was prepared using API-TOF Reference Mass Solution Kit (p/n: G1969-85001 (Santa clara, California, USA).Ten microliters of HP921 and 5 μL of purine were dissolved in one liter of methanol:acetonitrile:water (750:200:50) containing 0.1% acetic acid and were sprayed using an isocratic pump at a flow rate of 0.4 mL/min. The chromatographic parameters are shown in Table 2.

### 2.6. GC/Q-TOF Conditions

The derivatization and experimental parameters for both aqueous and organic fractions were performed as described elsewhere [19]. Agilent 7200 GC/Q-TOF (Santa clara, CA, USA) was used for acquisition with absolute retention times, which was locked to the internal standard d27 myristic acid from the Agilent Fiehn GC/MS Metabolomics Standards Kit (Part Number 400505; (Santa clara, CA, USA).with a retention time locking (RTL) software system. The GC/Q-TOF conditions used are provided in Table 3. 

### 2.7. Data Analysis

Agilent MassHunter Qualitative Analysis (version B.06.00 SP1) software (Santa clara, CA, USA) was used for processing MS and AutoMSMS data acquired using LC/GC/Q-TOF. The accurate mass MS data were processed using the tool “Find by Molecular Feature” to export the compounds to Agilent Mass Profiler Professional (MPP) software (Santa clara, CA, USA). In order to remove the molecular features arising from the background, the data obtained from each fraction were background-subtracted using the blank data again in MPP. The ID browser software was used to identify putative compounds by searching against the METLIN database (MassHunter PCDL Manager version B.04.00), which has 64,092 compounds. The GC/Q-TOF data were processed using MassHunter Unknown Analysis software (version B.06.00(Santa clara, CA, USA)). This software uses mass spectral deconvolution, which automatically finds peaks and deconvolutes spectra from co-eluting compounds using model ion traces. The spectral information was matched with the Agilent Fiehn library with retention time index with respect to FAME mix (Agilent Fiehn GC/MS Metabolomics Standards Kit, Part Number 400505). The data were also searched against NIST 11 and Wiley 9 mass spectral libraries. The compounds with library match scores >70% were considered and were searched in the literature for their therapeutic importance.

The LC-MS/MS data were processed by using the tool “Find by AutoMSMS”, and the spectral pattern generated was searched against the METLIN metabolite library, comprising accurate mass MS/MS information for 19,714 compounds. A few selected compounds found in *Alangium* species that were detected in METLIN database but lacked entry in METLIN library were processed using Molecular Structure Correlator software (MSC) (Santa clara, CA, USA). The MSC software (version B.05.00 build 19) performs systematic in silico bond breaking for the proposed structure and matches with the observed fragment ions, followed by assignment of an overall score. Here, the interface provided visualizations of the formula and the overall score, which was combined from the MS and MSMS score, along with the molecular formulas for the fragment ions with ppm *m/z* error.

## 3. Results and Discussion

**Figure 2:**
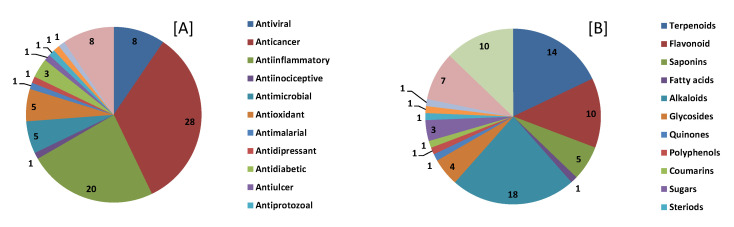
METLIN database matched compounds from *Alangium salviifolium* and grouped by therapeutic use (**A**) and compound class (**B**) based on literature reports.

In this study, we performed a comprehensive analysis of *A. salviifolium* bark metabolites using multi-separation protocols/ionization modes and multi-platform approaches. Initially, we performed fraction collection of the aqueous and organic extracts by injecting 1 mL of the extract for preliminary separation and enrichment of the metabolites. The Accurate Mass MS results when matched with METLIN database tentatively found 954 compounds with database match score >90%. Literature search revealed 81 of 954 compounds have therapeutic properties. The majority of these therapeutic compounds were secondary metabolites that are reported to have anti-cancer and anti-inflammatory activities (Figure 2). These compounds belonged to various plant secondary metabolite classes such as terpenoids, flavonoids, saponins, alkaloids, glycosides, etc. AutoMSMS analysis of all fractions resulted in identification of 449 compounds.

**Figure 3:**
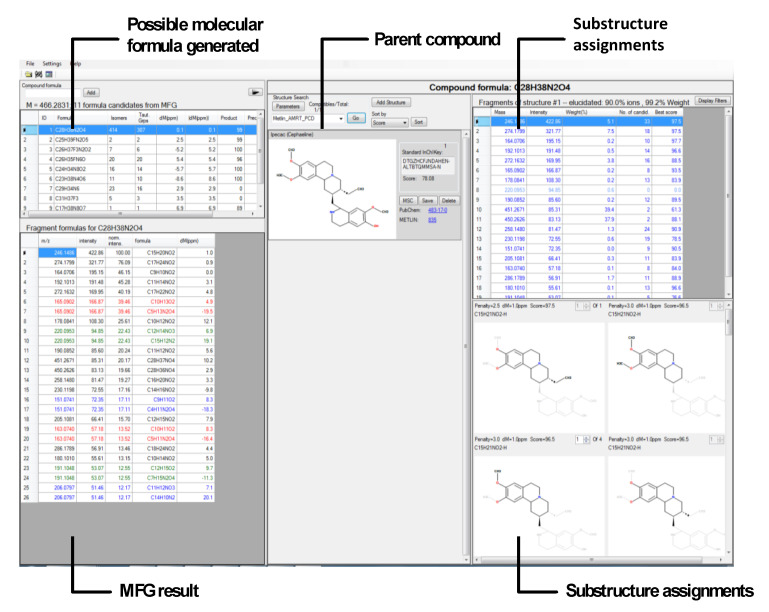
Results from Agilent MSC software tool for identifying the compounds that did not have a spectral match in the METLIN MSMS library.

**Figure 4:**
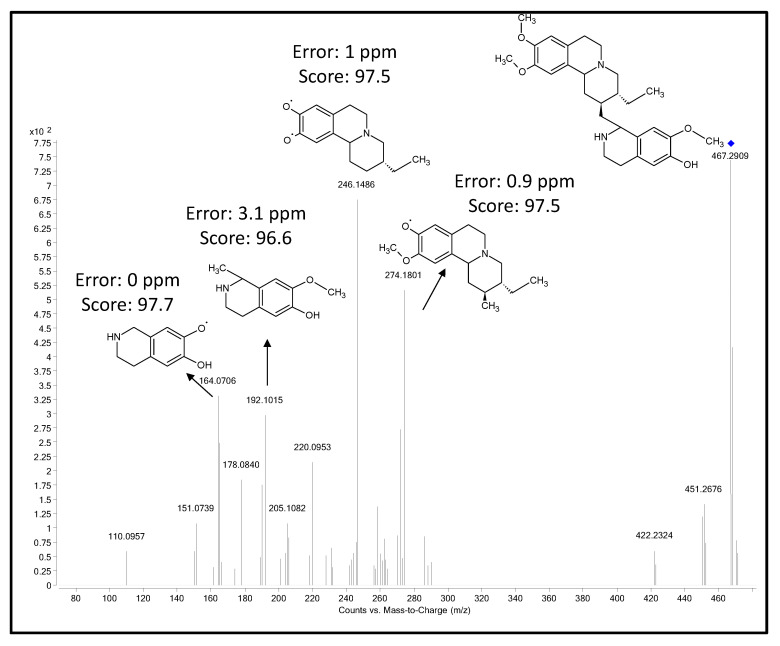
Proposed metabolite fragment structures for cephaeline based on MSC analysis.

Five compounds reported to be present commonly in *Alangium* species could not be identified in this study by LC-MS/MS spectral matching, since the spectra for these compounds were not available in the METLIN MSMS library. The spectral information was used to identify the compounds by using Agilent MassHunter MSC software (Figure 3). The overall MSC score for all the compounds was >97%, except for cephaeline which was 80%. Using the accurate mass precursor and fragment ion information for cephaeline, and the METLIN accurate mass database, we were able to identify the putative structures based on the MSMS spectra obtained for cephaeline (Figure 4). Thus, using MSC for tentative ID confirmation can be a useful tool in shortlisting the number of compounds for subsequent confirmation using actual standards. Table 4 shows the *Alangium* compounds identified by MSC software.

**Figure 5:**
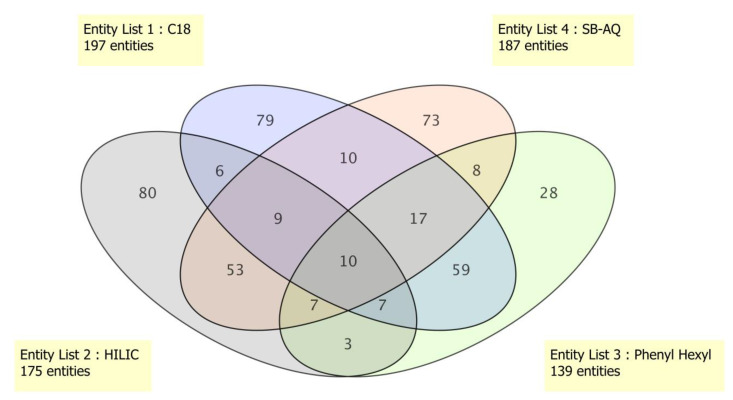
METLIN library matched compound distribution based on column chemistry. Figure drawn using MPP Software.

The separation chemistries for LC-MS/MS were performed using HILIC and three orthogonal reverse phase columns (ZORBAX Eclipse Plus C18, SB-AQ, and Phenyl Hexyl) for the separation of hydrophilic and hydrophobic compounds, respectively. The maximum number of compounds were identified in C18 (197) followed by SB-AQ (187), HILIC (175), and Phenyl Hexyl (139) columns (Figure 5). Significant compound overlaps were found between HILIC/SB-AQ and C18/Phenyl Hexyl columns: 53 and 59 compounds, respectively. Only 10 compounds were common to all 4 column types. The three different reverse phase columns, namely C18, SB-AQ, and Phenyl Hexyl, separated 79, 73, and 28 unique compounds, respectively, and HILIC revealed 80 unique compounds. Similar observations on enhanced metabolite coverage have been made by using HILIC and a reverse phase C18 column [20]. Our results using three different RP columns (for non-polar and intermediate polar), along with a HILIC (for polar compounds), clearly reveal the requirement for different separation chemistries for uncompromised metabolomics study.

**Figure 6:**
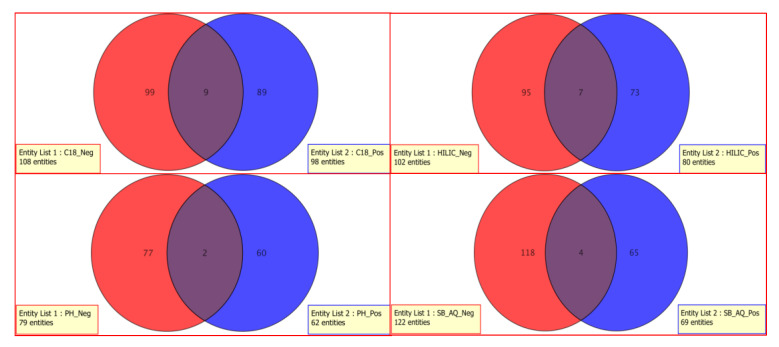
METLIN library matched compounds distribution based on ionization modes.

The compounds obtained from MS/MS analyses in positive and negative ionization modes are summarized in Figure 6. Clear differentiation of compounds for both ionization modes was observed for all the column chemistries used in the study. Many sugars and acidic amino acids were detected by negative mode ionization compared to positive mode ionization. Less than nine compounds were common for positive and negative ionization modes among all the column types. This clearly reveals that use of single ionization mode could significantly reduce the coverage of metabolites.

**Figure 7:**
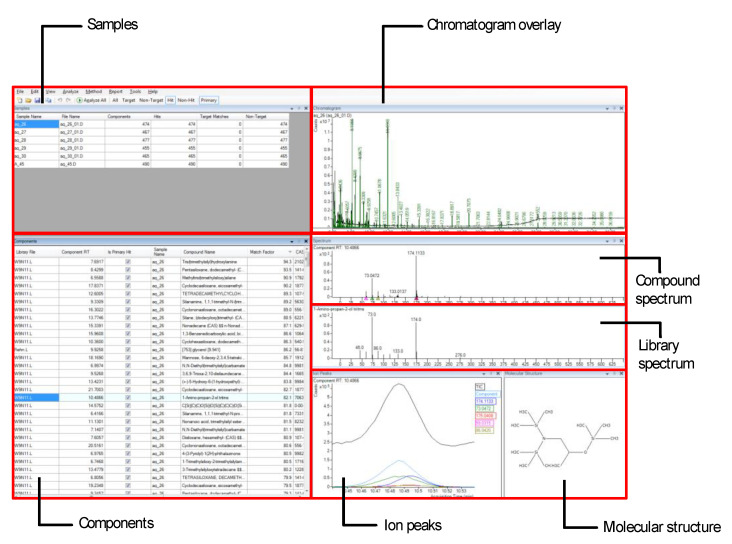
Fiehn/Wiley/NIST library matched analysis using Agilent MassHunter Unknown Analysis software.

The screen shot of unknown analysis software is shown in Figure 7. This software provides the features for visualization of the chromatograms, spectrum comparison, query vs. database spectrum alignment, molecular structure, and the components. The components comprise details for each compound.

**Figure 8:**
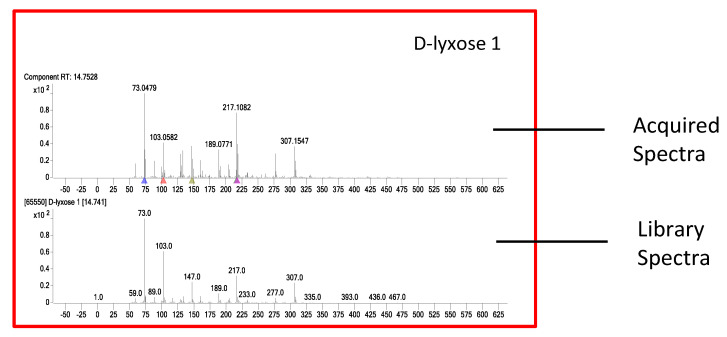
GC/Q-TOF spectral search results.

The compounds found by GC/Q-TOF were mostly flavonoids, fatty acids, sugars, terpenes, etc. As an example, D-lyxose identified from GC/Q-TOF analysis of aqueous extract using MassHunter Unknown Analysis software is shown in Figure 8. The top trace is the acquired GC/Q-TOF spectra, while the bottom trace is the Fiehn library spectra. The matching score was89.6. In addition, the retention time (RT) in the library (14.74 min) matched the RT of the acquired spectra (14.75 min).

**Figure 9:**
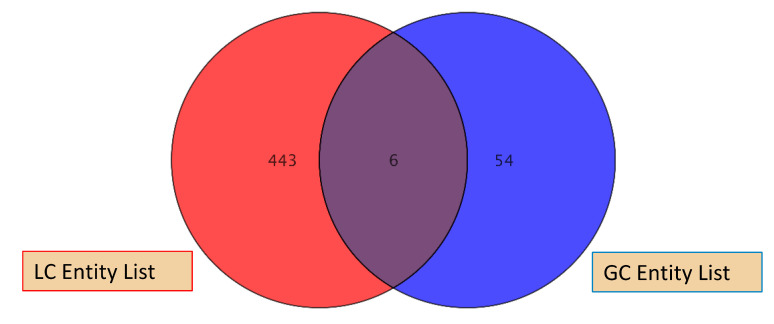
Compounds identified usingLC/Q-TOF and GC/Q-TOF analyses.

The LC/Q-TOF and GC/Q-TOF analysis resulted in identification of 449 and 62 compounds, respectively. The enhanced number of compounds observed for LC/Q-TOF was primarily due to the use of orthogonal separations. It is well established that LC/MS and GC/MS are complementary techniques for comprehensive metabolomics in order to identify non-volatile and volatile compounds (Figure 9).

## 4. Conclusions

This study demonstrates the utility of applying a comprehensive metabolite separation and detection strategy to aid in identification of metabolites in *A. salviifolium* bark. A multi-platform approach was used to detect compounds with different degrees of polarity. In addition, fractionation was used for enrichment, as well as four different column chemistries, along with two ionization modes for increasing the total number of metabolites identified. The compounds not found in the METLIN library were identified by using MSC software. Eighty-one secondary metabolites, such as ankorine, deooxytubulosine, ipecac, lacinilene C7-methyl ether, tubulosine, etc., which were identified in this study, are reported to have therapeutic value.

## Figures and Tables

**Table 1:** Chromatographic parameters for fractionation.

Parameters	Aqueous Extract		Organic Extract	
Mobile phase	Mobile phase A: Water + 10 mM ammonium acetate Mobile phase B: 100% Acetonitrile		Mobile phase A: 95:5 Water:methanol with 0.1% formic acid and 5 mM ammonium formate Mobile phase B: 65:30:5 Isopropanol:methanol:water with 0.1% formic acid and 5 mM ammonium formate	
Flow rate	1.2 mL/min		1.2 mL/min	
Injection volume	1 mL		0.3 mL	
Thermostat autosampler	4 °C		4 °C	
Temperature TCC	25 °C		25 °C	
DAD	210 and 254 nm		210 and 254 nm	
Peak width	>0.05 min		>0.05 min	
Fraction collection mode	Time based		Time based	
Total time	13 min		13 min	
Column	ZORBAX SB-C18(9.4 × 50 mm, 5 µm, p/n: 846975-202)		ZORBAX SB-C18(9.4 × 50 mm, 5 µm, p/n: 846975-202)	
Time slices	0.292 min/well		0.292 min/well	
Gradient	Time (min) 0.0 1.0 8.0 8.1 10.0 10.1 12.0	% Solvent B 5 5 35 95 95 5 5	Time (min) 0.0 1.0 8.0 11.0 11.1 12.0	% Solvent B 60 60 100 100 60 60

**Table 2:** Chromatographic parameters used in the LC/MS and LC-MS/MS analysis.

Parameter	Aqueous Fractions Analysed Using ZORBAX SB-AQ Column		Aqueous Fractions Analysed Using ZORBAC HILIC Column	
Ionization mode	Positive MS and positive AutoMSMS	Negative MS and Negative AutoMSMS	Positive MS and positive AutoMSMS	Negative MS and Negative AutoMSMS
Mobile phase	Mobile phase A: Water with 0.2% acetic acid Mobile phase B: Methanol with 0.2% acetic acid	Mobile phase A: Water with 1 mM ammonium fluoride acetic acid Mobile phase B: 100% Acetonitrile	Mobile phase A: 90:10 of Acetonitrile: 50 mM ammonium acetate Mobile phase B: 50:40:10 of acetonitrile:water:50 mM ammonium acetate	Mobile phase A: 90:10 of Acetonitrile:50 mM ammonium acetate Mobile phase B: 50:40:10 of acetonitrile:water:50 mM ammonium acetate
LC gradient	Time (min)	% mobile phase B	Time (min)	% mobile phase B
	1.00	5.0	3.00	0.0
	10.0	35.0	10.0	100.0
	11.0	95.0	13.0	100.0
	13.0	95.0	13.10	0
	13.1	5.0	17.00	0
	15.0	5.0		
Parameter	Organic fractions analysed using ZORBAX EP-C18		Organic fractions analysed using ZORBAX EP Phenyl Hexyl	
Ionization mode	Positive MS and positive utoMSMS	Negative MS and Negative AutoMSMS	Positive MS and positive AutoMSMS	Negative MS and Negative AutoMSMS
Mobile phase	95:5 of water: Methanol with 0.1% formic acid and 5 mM ammonium formate	65:30:5 of Isopropanol:methanol: water with 0.1% formic acid and 5 mM ammonium formate	95:5 of water: Methanol with 0.1% formic acid and 5 mM ammonium formate	65:30:5 of Isopropanol:methanol: water with 0.1% formic acid and 5 mM ammonium formate
LC gradient	Time (min)	% of mobile phase B	Time (min)	% of mobile phase B
	1.00	60.0	1.00	60.0
	8.0	100.0	8.0	100.0
	11.0	100.0	11.0	100.0
	11.10	60.0	11.10	60.0
	14.00	60.0	14.00	60.0

**Table 3:** Conditions used for GC/Q-TOF.

**GC Conditions**	
Column	DB-5 ms: 30 m × 0.25 mmID × 0.25 μm, Guard Length: 10 m (Part No. 122-5532G)
Injection volume	1 µL
Split mode and ratio	Split 10:1
Split/Splitless inlet temperature	250 °C
Oven temperature program	60 °C for 1 min 10 °C/min to 325 °C, 10 min hold
Carrier gas	Helium at 1.2798 mL/min constant flow
Transfer line temperature	290 °C
**QTOF Conditions**	
Ionization mode	EI
Source temperature	230 °C
Quadrupole temperature	150 °C
*m/z* scan	50 to 600 *m/z*
Spectral acquisition rate	5 spectra/s, 2679 transients/spectrum, collecting both in centroid and profile modes

**Table 4:** Compounds found in *Alangium* species identified by Agilent MSC Software.

Metabolite	CAS/KEGG	Formula	Mass Difference (ppm)	Overall Score	Confirmed by MSC
Ankorine	13849-54-2	C19 H29 N O4	−2.56	99.94	YES
Deoxytubulosine	C11817	C29 H37 N3 O2	−0.69	98.97	YES
Ipecac (Cephaeline)	483-17-0	C28 H38 N2 O4	−0.28	80.06	YES
Lacinilene C 7-methyl ether	56362-72-2	C16 H20 O3	−1.84	98.95	YES
Tubulosine	2632-29-3	C29 H37 N3 O3	2.86	97.31	YES
